# Differential Expression Profile and Genetic Variants of MicroRNAs Sequences in Breast Cancer Patients

**DOI:** 10.1371/journal.pone.0030049

**Published:** 2012-02-20

**Authors:** Ali A. Alshatwi, Gowhar Shafi, Tarique N. Hasan, Naveed Ahmed Syed, Amal A. Al-Hazzani, Mohammed A. Alsaif, Abdulaziz A. Alsaif

**Affiliations:** 1 Molecular Cancer Biology Research Lab (MCBRL), Department of Food Sciences and Nutrition, College of Food and Agricultural Sciences, King Saud University, Riyadh, Saudi Arabia; 2 Department of Botany and Microbiology, College of Science, King Saud University, Riyadh, Saudi Arabia; 3 College of Applied Medical Sciences, King Saud University, Riyadh, Saudi Arabia; 4 College of Medicine, King Saud University, Riyadh, Saudi Arabia; East Carolina University, United States of America

## Abstract

The technology available for cancer diagnosis and prognosis is not yet satisfactory at the molecular level, and requires further improvements. Micro RNAs (miRNAs) have been recently reported as useful biomarkers in diseases including cancer. We performed a miRNA expression profiling study using peripheral blood from breast cancer patients to detect and identify characteristic patterns. A total of 100 breast cancer patients and 89 healthy patients were recruited for miRNA genotyping and expression profiling. We found that hs-miR-196a2 in premenopausal patients, and hs-miR-499, hs-miR-146a and hs-miR-196a2 in postmenopausal patients, may discriminate breast cancer patients from healthy individuals. In addition, we found a significant association between two microRNA polymorphisms (hs-miR-196a2 and hs-miR-499) and breast cancer risk. However, no significant association between the hs-miR-146a gene and breast cancer risk was found. In summary, the study demonstrates that peripheral blood miRNAs and their expression and genotypic profiles can be developed as biomarkers for early diagnosis and prognosis of breast cancer.

## Introduction

Breast cancer incidence shows geographical variation, even in ethnically homogeneous areas. Over the past few years, Saudi Arabia has witnessed an increase in the occurrence of breast cancer in its population. The increase could be a result of exposure to environmental carcinogens, different lifestyles and reproductive patterns, or differences in dietary or cultural practices of Saudi women. In Saudi Arabia, breast cancer constitutes 18% of all cancers in Saudi women [1]. Although locally advanced breast tumor is unusual in Western countries, it constitutes more than 40% of all non-metastatic breast cancer in Saudi Arabia. In the Saudi population, 21% of all female cancer diagnoses are breast cancer. Age-adjusted breast cancer incidence rates in Western countries are about five times higher than rates in less developed countries [1]. However, in the 2002 annual report of the Saudi National Cancer Registry, 26.4% of all female breast cancers in Saudi Arabia develop before the age of 40, as compared with only 6.5% in the USA. It has been reported that young age is an independent risk factor for female breast cancer in the Saudi population [2].

MicroRNAs (miRNAs) are short (∼19–23 nucleotides), non-coding RNA molecules that are recognized as endogenous physiological regulators of gene expression. These small RNAs are capable of controlling gene expression either by repressing translation and transcription [3], or by activating transcription [4]. MiRNAs are also known to play important roles in many physiological and pathological processes, including tumorigenesis [5], proliferation [6], hematopoiesis [7], metabolism [8], immune function [9], epigenetics, and neurological diseases [10], [11]. They were also found to be useful in identifying lymphoma causes [12] and the progression of certain neurological diseases [13].

According to UCSC genome browser (http://genome.ucsc.edu), several SNPs falling within miRNA hairpin regions are reported and are likely to have an expected reduced biological effect, because they do not change substantially the structure of the miRNA. Since miRNAs function as oncogenes or tumor suppressors [3]–[5] altered expressions of miRNAs may contribute to prostatic malignant transformation. Therefore, we hypothesized that SNPs in miRNAs may be associated with breast cancer risk. We evaluated the association of three selected SNPs (rs11614913, rs2910164, and rs3746444), which are located at the miRNA hairpin regions of, hsa-mir196a2, hsa-mir146a, and hsamir499, respectively with breast cancer risk in a case–control study. A single nucleotide polymorphism (SNP) located in the miRNA binding site of a miRNA target may disrupt miRNA-target interaction, resulting in the deregulation of target gene expression, as shown in non-small cell lung cancer [14]. Alternatively, a SNP within the miRNA precursor may alter miRNA processing and ultimately change the mature miRNA level, as was recently shown for miR-146a in papillary thyroid carcinoma [15]. Such effects by SNP on miRNA levels and function may modify breast cancer risk in general, and may explain the different risk level for Saudi women.

Serum/plasma miRNAs derived from various tissues/organs are stable and resistant to nuclease digestion. Therefore, examination of miRNA expression levels in blood is reproducible and is indicative of the disease state [16]. We hypothesized that specific miRNA signatures in blood samples can be used to identify biomarkers for diagnosis, prognosis or even etiology of a disease. In this study, we show that disease progression and the breast cancer stages could be identified from miRNA profiles, when comparing blood samples from breast cancer patients with those from healthy patients.

## Results

### Characteristics of patients

The clinicopathologic records for each patient were published previously [17] (Table 1). The average age of the randomly selected group of 100 breast carcinoma cases with stage I (24%), stage II (39%), stage III (33%), and stage IV (2%) and two patients undetermined disease was about 51.5 years. Fifty-eight percent of the patients were premenopausal cases, with an average age of 37.5 years. The average age of postmenopausal patients was 61.2 years.

**Table 1:** Characteristics of breast cancer patients.

	Age group Characteristics		Tumor stage			
	Premenopausal (average age 37.5years)	Postmenopausal (average age 61.2years)	I	II	III	IV
No. of patients (N = 100)	58	42	13	50	35	2

### Expression of hs-miR-196a2, hs-miR-499 and hs-miR-146a in blood from breast cancer patients and healthy patients

**Figure 1:**
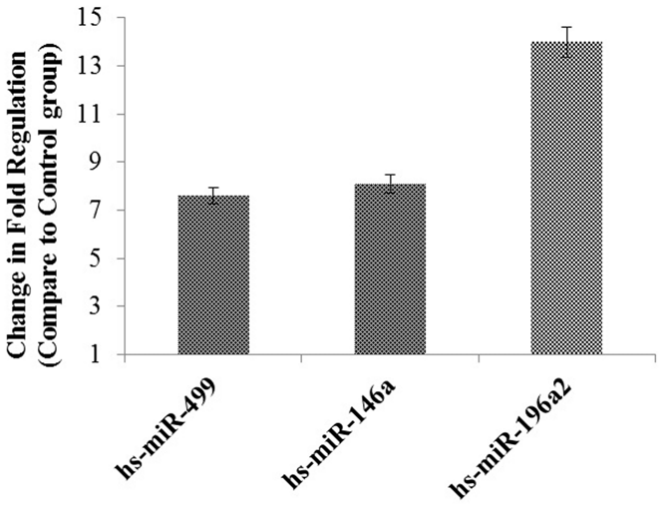
Relative expression of microRNAs in breast cancer cases and control subjects. Total RNA including small RNA was isolated from blood samples from healthy subjects and breast cancer patients using the Blood RNA Isolation Kit (Qiagen, Germany). cDNA was subjected to Real Time PCR using microRNAs assays (Qiagen, Germany). Data derived from quantitative real-time PCR and presented in ΔCT of relative threshold cycles indicating fold changes over normal subjects. Normalization was performed with the small nuclear RNU1A in blood samples. Results are mean values of triplicate experiments. Bars denote standard error (SEM). P-values of the statistical evaluations of miRNA levels were determined by Mann and Whitney-U test.

**Figure 2:**
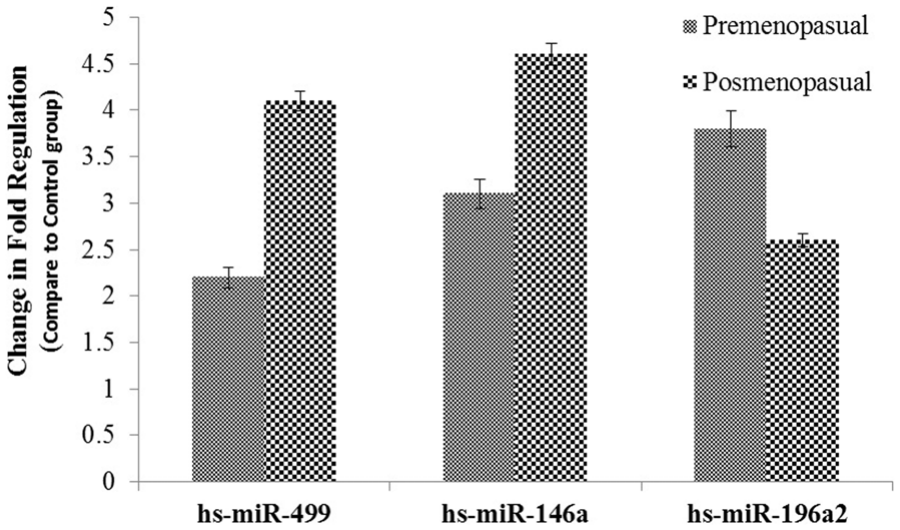
Relative expression of microRNAs in pre- and post-menopausal breast cancer patients. Total RNA including small RNA was isolated from blood samples from healthy subjects and breast cancer patients using the Blood RNA Isolation Kit (Qiagen, Germany). cDNA was subjected to Real Time PCR using microRNAs assays (Qiagen, Germany). Data derived from quantitative real-time PCR and presented in ΔCT of relative threshold cycles indicating fold changes over normal subjects. Normalization was performed with the small nuclear RNU1A in blood samples. Results are mean values of triplicate experiments. Bars denote standard error (SEM). P-values of the statistical evaluations of miRNA levels were determined by Mann and Whitney-U test.

Numerous publications have reported aberrant miRNA expression in cancer. Figure 1 shows the relative quantification of miRNAs hs-miR-196a2, hs-miR-499 and hs-miR-146a in the blood of 89 healthy individuals and 92 patients with breast cancer (RNA quality or quantity was insufficient for expression study in 8 out of the 100 samples, which will be the subject of future investigation; thus the final analyses included 92 cases out of the 100). To determine the differences in the relative expression profiles, we performed univariate analyses using the Mann Whitney-U test. Quantitative reverse transcriptase-polymerase chain reaction assays confirmed this finding. As shown in Figure 2, the median values of hs-miR-196a2 for the pre- and post-menopausal subgroups were increased 3.8-fold (*P* = 0.0001) and 2.6-fold (*P* = 0.008) respectively, in comparison to healthy individuals. In contrast, the values for hs-miR-499 (4.1-fold, *P* = 0.005) and hs-miR-146a (4.6-fold, *P* = 0.001) could only discriminate the disease state in postmenopausal patients, but not in premenopausal patients, when compared with healthy individuals.

### MicroRNA genotyping

Tables 2 and 3 show the genotypic frequencies of the three polymorphic miRNA genes from genomic DNA extracted from breast cancer cases and in controls. Significant association was found between the two polymorphisms hs-miR-196a2 (rs11614913) and hs-miR-499 (rs3746444) and breast cancer risk. However, no significant association between hs-miR-146a (rs2910164) and breast cancer risk was found. For hs-miR-196a2, the heterozygous genotype (CT) showed risk for disease (*P* = 0.01; OR = 1.70). In addition, the variant allele carrier (CT+TT) also displayed a higher breast cancer frequency (65%) than controls (54%), showing a risk pattern for breast cancer (*P* = 0.01; OR = 1.66). Similarly, for the hs-miR-499 polymorphism, we found that the heterozygous genotype (CT) conferred a much higher risk for developing breast cancer (*P* = 0.001; OR = 2.27). The variant allele carrier (CT+CC) also showed a higher risk pattern for breast cancer, with breast cancer cases (70%) and controls (55%) (*P*≤0.001; OR = 1.97). The allelic frequencies did not show any associations for the three polymorphisms (Table 4). Furthermore, PCR products were randomly selected from the real-time master plate and were subjected to electrophoresis on 2% agarose gel to confirm the genotype of the three microRNA genes under study.

**Table 2:** Patients showing microRNA genetic variants categorized by tumor stage (*n*).

Tumor stage	+/+	+/−	−/−
**hs-miR-196a2**			
I	3	9	1
II	14	22	14
III	7	13	7
IV	1	0	1
Undermined	8		
**hs-miR-499**			
I	4	2	7
II	4	28	11
III	15	12	7
IV	1	0	1
Undermined	8		
**hs-miR-146a**			
I	5	7	0
II	14	21	15
III	8	7	11
IV	2	0	0
Undermined	8		

**Table 3:** Distribution of microRNA gene genetic variants and Odds ratio with 95% CI in breast cancer patients and control subjects.

Genetic variants	Control	Patients	OR (95% CI)	P value
**hs-miR-499**				
Wild type [+/+]	45	30	1^ref^	
Heterozygous [+/−]	40	62	2.22(1.40–3.52)	0.001
Homozygous [−/−]	15	8	0.79(0.35–1.69)	0.56
Recessive Model	55	70	1.92(1.23–3.0)	0.001
**hs-miR-196a2**				
Wild type [+/+]	46	35	1^ref^	
Heterozygous [+/−]	50	63	1.65 (1.05–2.55)	0.01
Homozygous [−/−]	4	2	0.45(0.07–1.86)	0.25
Recessive Model	54	65	1.61(1.03–2.44)	0.01
**hs-miR-146a**				
Wild type [+/+]	51	48	1^ref^	
Heterozygous [+/−]	46	50	1.06(0.68–1.63)	0.51
Homozygous [−/−]	3	2	0.77(0.18–2.89)	0.71
Recessive Model	49	52	1.11(0.72–1.70)	0.41

**Table 4:** Distribution of microRNA gene allelic variants and Odds ratio with 95% CI in breast cancer patients and control subjects.

Allelic variants	Control	Patients	OR (95% CI)	P value
**hs-miR-499**				
T	130	122	1^ref^	
C	70	78	1.14(0.84–1.56)	0.18
**hs-miR-196a2**				
C	142	133	1^ref^	
T	58	67	1.18(0.85–1.62)	0.18
**hs-miR-146a**				
C	148	146	1^ref^	
G	52	54	1.01(0.71–1.40)	0.7

### Association of microRNA expression with microRNA gene variants

**Figure 3:**
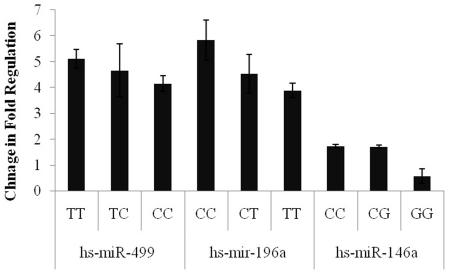
Relative expression of microRNAs with respect to genotype pattern. miRNAs were determined using SYBR Green MicroRNA Assays adopting Quantitative Real Time PCR. Normalization was performed with the small nuclear RNU1A in blood samples. Total RNA including small RNA was isolated from blood samples from healthy subjects and breast cancer patients using the Blood RNA Isolation Kit (Qiagen, Germany). cDNA was subjected to Real Time PCR using microRNAs assays (Qiagen, Germany). Data derived from quantitative real-time PCR and presented in ΔCT of relative threshold cycles indicating fold changes over normal subjects.Results are mean values of triplicate experiments. Bars denote standard error (SEM). P-values of the statistical evaluations of miRNA levels were determined by Mann and Whitney-U test.

To further investigate the functional relevance of miRNA gene polymorphism, correlation analysis between genotypes and miRNA expression was performed (Figure 3). We found that among the 92 breast cancer patients, 25 were of the rs11614913 TT genotype (hs-miR-196a2), 44 of the CT genotype, and 23 of the CC genotype. The Δ_Ct_ value for hs-miR-499 of the rs3746444 TT genotype was 5.12±0.36; the CT genotype was 4.66±1.03; and the CC genotype was 4.16±0.3, whereas Δ_Ct_ values for mature *hs-mir-196a* according to different rs11614913 genotypes were 5.83±0.77 (24), 4.53±0.75 (42), and 3.89±0.28 (26), respectively. However, the Δ_Ct_ value for the hs-miR-146a miRNA (rs2910164) TT genotype was 1.74±0.07 (29); the CT genotype was 1.72±0.06 (35), and 0.59±0.28 (26) for the CC genotype (*P = *0.437).

## Discussion

In this study, we examined whether the expression profile of three miRNAs (hs-miR-196a2, hs-miR-499 and hs-miR-146a) in blood were associated with the diagnosis and progression of breast cancer, based on the regulation of relevant target genes. The present study reveals that hs-miR-196a2 may discriminate breast cancer patients from healthy individuals in premenopausal patients, while hs-miR-499, hs-miR-146a and hs-miR-196a2 may be relevant in postmenopausal patients. Recent studies have shown that miRNA-binding SNPs regulate oncogenes, tumor suppressor genes, or genes that contribute to the carcinogenesis [18]. Yu et al. [19] performed a genome-wide analysis of SNPs within the 3′-UTR miRNA binding sites of various human genes, and found that there was significantly different allele frequencies of some miRNA-binding SNPs reported in human cancer expressed sequence tag libraries and the National Center for Biotechnology Information SNPs database [20], suggesting that certain SNPs located within miRNA binding sites may be over-represented in individuals with cancer.

To the best of our knowledge, this is the first study to provide evidence that common SNPs in miRNAs might play an important role in breast cancer prediction. Recently, a strong link between miRNAs that are altered, either structurally or in the number of mature miRNAs, and cancer has been demonstrated [21]. Mutations, polymorphisms, misexpression, or altered mature miRNA processing are likely to be pleiotropic and may contribute to cancer susceptibility and progression. Inherited mutations or rare SNPs in the primary transcripts of *has-mir-15a* and *has-mir-16-1* have been linked to familial chronic lymphocytic leukemia and familial breast cancer [22], which was further supported in chronic lymphocytic leukemia [23]. It has also been shown that miRNA expression patterns have relevance to the biological and clinical behavior of human solid tumors [24]–[26]. For example, *hsa-mir-196a* was highly expressed in pancreatic [25] and breast cancers [26] compared with normal tissues, and the elevated expression was associated with significantly reduced survival for pancreatic cancer [25].

In addition, the present study showed that hs-miR-196a2 heterozygous genotypes and variant allele carriers were significantly associated with 1.8- and 1.7-fold, respectively, higher risk for developing breast cancer. Similarly, hs-miR-499 was observed to be a 2.3-fold greater risk for patients carrying the heterozygous genotype, while variant allele carriers also revealed 2.0-fold risk pattern for breast cancer. On the other hand, hs-miR-146a was not found to be associated with the disease. Hence, in this population-based study of breast cancer in Saudi Arabia, it was observed that the genetic polymorphism in the hs-miR-196a2 (rs11614913) and hs-miR-499 (rs3746444) genes are associated with increased risk of developing breast cancer. On the contrary, the hs-miR-146a (rs2910164) gene was found to be a non-significant factor. To the best of our knowledge, this is the first study of the Saudi population to correlate miRNA polymorphism with breast cancer susceptibility. Though our study has certain limitations in terms of the non-significant association of the alleles with breast cancer risk with reference to disease development, nevertheless it has several strengths too. The control subjects followed the Hardy-Weinberg equilibrium, all of our cases were histopathologically confirmed and proper guidelines for ensuring the quality of our genotyping were used. The non-significant association of the polymorphisms with clinical parameters could be due to the small sample size, or perhaps these polymorphisms do not have a role in tumor metastasis and progression.

Protein based biomarkers from whole blood, serum or plasma have been widely used over the years for clinical diagnosis and prognosis. MiRNA-induced gene expression has also been shown to contribute extensively to disease phenotype [27]. The apparent roles of miRNAs in diseases have led many researchers to probe the molecular mechanisms underlying pathogenesis and to develop novel diagnostic and therapeutic agents [28]. We observed that the miRNAs were stably expressed in circulation even several months after the onset of breast cancer. This correlated with the observation from Chen et al. [16] that miRNAs from serum are stable, resistant to nuclease digestion and almost consistent among individuals.

In conclusion, the results imply that individual and combined genotypes of microRNA processing pathway genes may influence breast cancer susceptibility. As a result, breast cancer-related miRNAs may represent novel biomarkers, as they are potentially useful in the diagnosis and prognosis, and the monitoring of treatment response. When these important miRNAs are identified and their functions elucidated, we can manipulate them for therapeutic benefit in a rational manner. The study provides the first epidemiologic evidence evaluating the effects of genetic polymorphisms in pre-microRNA genes with breast cancer risk. These findings propose that breast cancer-related miRNAs may eventually be exploited as therapeutic targets. Large cohort and diverse ethnicity studies will be required to validate our results. This would assist in a deeper understanding of the basic functions and pathological alterations in microRNA needed to pave the way for future clinical applications. Our findings suggest that genetic variations in miRNA-regulated genes are important screening tools for identifying individuals at high risk for developing breast cancer, particularly at an early age. However, larger studies with multi-ethnic groups are warranted to validate our findings.

## Materials and Methods

### Clinical methodology

A total of 100 breast cancer patients admitted to the King Khalid University Hospital were selected for the study. The study protocol included a standard oncological evaluation with subsequent review and outpatient follow-ups. In our study, we used blood samples collected from breast cancer patients and healthy volunteers. Demographic data, medical history and conventional vascular risk factors were recorded in a standardized computerized database and extracted from the medical records. The study was approved by the hospital ethical committee board. Written informed consent was obtained from all patients and control subjects included in this study.

### DNA extraction

Genomic DNA was extracted using the QIAamp DNA blood mini kit (Qiagen, Hilden, Germany), as per manufacturer's instructions.

### MicroRNA extraction

Total RNA, including microRNA, was extracted from blood samples using the Blood RNA isolation kit (Qiagen, Hilden, Germany) as per manufacturer's instructions. RNA concentration was quantified using NanoDrop ND-1000 Spectrophotometer (Thermo Fisher Scientific, USA). RNA quality was determined using a 2% agarose gel.

### Genotyping

SNP analysis was performed using a real-time PCR (RT-PCR) allelic discrimination TaqMan assay (Applied Biosystems, Foster City) with slight modifications. The assay included proprietary non-labeled forward and reverse primers along with two proprietary fluorescent TaqMan oligonucleotide probes (allele 1-specific probe labeled with VIC fluorophore, allele 2-specific probe labeled with FAM (6-carboxy- fluorescein fluorophore). All PCRs were performed in duplicate. The reaction contained 50 ng (10 µL) DNA, 9 µL TaqMan genotype PCR Master Mix (2×), and 1 µL allelic discrimination mix. RT-PCR was performed using ABI Prism 7500 Fast Sequence Detection System (SDS; Applied Biosystems, Foster City, California, USA) using the following conditions: 50°C for 2 min, 95°C for 10 min, and then 40 cycles of amplification (92°C denaturation for 15 s, 62°C annealing/extension for 60 s). The annealing temperature was empirically determined (data not shown) to promote high probe specificity without losing assay sensitivity. For each cycle, the SDS software determined the ΔRn, which is the normalized (i.e., compared with a passive reference fluorophore) fluorescent signal from the VIC- or FAM-labeled probe. For our analysis, we used the ΔRn value after the final cycle because it proved more reliable than the cycle at which the threshold was crossed.

### cDNA synthesis and microRNA real-time PCR

MiRNA quantitation was determined using SYBR Green PCR. Briefly, 100 ng of template RNA was reverse transcribed in 20 µL using a universal stem-loop primer to generate the RT-product. For the PCR reaction, 20 ng of RT-product was used. PCR was performed using the Applied Biosystems 7500 Fast Sequence Detection System. PCR reactions were performed in triplicate, in three separate experiments. miRNAs were considered present when the Ct values (threshold cycle) were lower than 35. RNU1A was used as the normalization gene.

### Statistical analysis

Statistical analyses were performed using the SPSS software package, version 18.0 (SPSS Inc. Chicago, IL, USA). The chi-square or the two-tailed Fischer's exact test was used to identify potential associations of miRNA concentrations in blood with clinical and histopathological risk factors in breast cancer patients. The cycle threshold (Ct) value for the genes was determined using SDS software v1.2 (Applied Biosystems, Foster City, California, USA). MiRNA expression level was normalized to the internal control RNU1A. The relative gene expression was calculated as 2^−ÄCt^. The miRNA expression change observed in patients in relation to control group was determined using the 2^−ÄÄCt^ method. Statistical significance was determined using the Student's *t*-test and was considered significant if *P*<0.05.
